# The Myth of Cultured Meat: A Review

**DOI:** 10.3389/fnut.2020.00007

**Published:** 2020-02-07

**Authors:** Sghaier Chriki, Jean-François Hocquette

**Affiliations:** ^1^ISARA, Agroecology and Environment Unit, Lyon, France; ^2^INRAE, University of Clermont Auvergne, Vetagro Sup, UMR Herbivores, Saint-Genès-Champanelle, France

**Keywords:** cultured meat, *in vitro* meat, muscle cells, livestock farming, consumer perception, vegetarian, ethics

## Abstract

To satisfy the increasing demand for food by the growing human population, cultured meat (also called *in vitro*, artificial or lab-grown meat) is presented by its advocates as a good alternative for consumers who want to be more responsible but do not wish to change their diet. This review aims to update the current knowledge on this subject by focusing on recent publications and issues not well described previously. The main conclusion is that no major advances were observed despite many new publications. Indeed, in terms of technical issues, research is still required to optimize cell culture methodology. It is also almost impossible to reproduce the diversity of meats derived from various species, breeds and cuts. Although these are not yet known, we speculated on the potential health benefits and drawbacks of cultured meat. Unlike conventional meat, cultured muscle cells may be safer, without any adjacent digestive organs. On the other hand, with this high level of cell multiplication, some dysregulation is likely as happens in cancer cells. Likewise, the control of its nutritional composition is still unclear, especially for micronutrients and iron. Regarding environmental issues, the potential advantages of cultured meat for greenhouse gas emissions are a matter of controversy, although less land will be used compared to livestock, ruminants in particular. However, more criteria need to be taken into account for a comparison with current meat production. Cultured meat will have to compete with other meat substitutes, especially plant-based alternatives. Consumer acceptance will be strongly influenced by many factors and consumers seem to dislike unnatural food. Ethically, cultured meat aims to use considerably fewer animals than conventional livestock farming. However, some animals will still have to be reared to harvest cells for the production of *in vitro* meat. Finally, we discussed in this review the nebulous status of cultured meat from a religious point of view. Indeed, religious authorities are still debating the question of whether *in vitro* meat is *Kosher* or *Halal* (e.g., compliant with Jewish or Islamic dietary laws).

## Introduction: Context Of Animal Farming Today

The global population, 7.3 billion today, is expected to surpass 9 billion by 2050. The Food and Agriculture Organization (FAO) has forecast that in 2050, 70% more food will be needed to fulfill the demand of the growing population, which is a great challenge due to resource and arable land limitations. Even if meat consumption is decreasing in developed countries, its global consumption is increasing because consumers are generally unwilling to reduce their meat consumption, in particular in developing countries such as in China, India, and Russia (1). These populations becoming more middle-class, they are looking for more luxury products, such as meat or other animal products (e.g., cheese, dairy products).

Livestock systems will contribute to addressing the issue of global food and nutrition security in the world (2). Animal farming must produce larger quantities of high quality and affordable meat, milk, and eggs, through production systems that are environmentally sound, socially responsible, and economically viable (3). Despite the wide range of economic, environmental, cultural and social services at local, regional, and global levels provided by livestock farming (4), a significant proportion of livestock is raised nowadays within the factory farming model. Despite a lower contribution to greenhouse gases (GHG) and water usage than extensive agriculture, factor farming is mainly focused on efficiency (i.e., the quantity of milk or meat produced) rather than on other services and impacts such as interaction with the environment, climate change, less use of antibiotics, animal welfare, or sustainability (5–8).

As a consequence, more efficient ways of protein production are being developed to sustain the growing global population while complying with today's challenges, such as environmental and animal welfare issues (9). Among the solutions, cultured meat is presented by its advocates as a sustainable alternative for consumers who want to be more responsible but do not wish to change the composition of their diet (10–13). The history of cultured meat was detailed by Hamdan et al. (14), and a bibliometric analysis of publications about this subject was carried out by Fernandes et al. (15). Indeed, since the first publication about cultured meat in 2008, the number of publications increased considerably (89% of the total) after 2013. In August of that same year, the first hamburger produced with cultured meat was prepared and tasted on a television program (16).

## The Production of Cultured Meat

### Pros and Cons of the Culture Process

The objective of this process is to recreate the complex structure of livestock muscles with a few cells. A biopsy is taken from a live animal. This piece of muscle will be cut to liberate the stem cells, which have the ability to proliferate but can also transform themselves into different types of cells, such as muscle cells and fat cells (16).

The cells will start to divide after they are cultured in an appropriate culture medium, which will provide nutrients, hormones and growth factors. The best medium is known to contain fetal bovine serum (FBS), a serum made from the blood of a dead calf, which is going to be rate-limiting, and not acceptable for vegetarians nor vegans. More than one trillion cells can be grown, and these cells naturally merge to form myotubes which are no longer than 0.3 mm; the myotubes are then placed in a ring growing into a small piece of muscle tissue as described in different reviews (17, 18). This piece of muscle can multiply up to more than a trillion strands (13). These fibers are attached to a sponge-like scaffold that floods the fibers with nutrients and mechanically stretches them, “exercising” the muscle cells to increase their size and protein content (17, 18). Based on this process, fewer animals will be necessary to produce huge amounts of meat due to cell proliferation, thereby avoiding killing as too many animals but potentially lots of calves if FBS is still used.

Throughout this process, the cells are kept in a monitored environment that replicates the temperature inside the body of a cow, for example, to speed up the development of the lab-grown meat (17, 18).

One initial problem with this type of culture is the serum used, as *in vitro* meat aims to be slaughter-free. So it is contradictory to use a medium made from the blood of dead calves. In addition, this serum is expensive and affects to a large extent the production cost of the meat. One of the main goals of the laboratory start-ups (about 25–30) as of this writing, scattered over the globe and working on cultured meat is to find a cheaper medium derived from plant ingredients and as efficient as FBS. Apparently (from personal communications), this problem has been solved, at least in research prototypes to produce cultured meat. Once this problem has been solved on an industrial scale (and it is likely to be solved), *in vitro* meat could become competitive in terms of production costs and animal ethics compared to regular meat from livestock. In addition to FBS, antibiotics and fungicides have been commonly used to avoid contamination of cell cultures. All the start-ups claim that this problem has also been solved.

However, as farm animals, like all mammals including humans, naturally produce hormones and growth factors to sustain their own growth, cell culture needs hormones, growth factors, etc., in the culture medium to sustain cell proliferation and differentiation. The research questions are now: how can these compounds be produced on an industrial scale, and how can be ensured that none of them will have negative effects on human health in the short and long term? This is an important issue since hormone growth promoters are prohibited in farming systems for conventional meat production in the European Union (unlike in some other parts of the world).

Finally, we are still far away from real muscle, which is made up of organized fibers, blood vessels, nerves, connective tissue and fat cells (19–21). This is why the different start-ups working in this area have developed different strategies: some of them work with stem cells or muscle cells to reproduce unorganized muscle fibers, which is the simplest approach, while others are trying to reproduce thin slices of muscles (i.e., muscle fibers and other cell types quite well imbricated together). Nevertheless, the production of a thick piece of meat like a real steak is still a dream, due to the necessity of perfusing oxygen inside the meat to mimic the diffusion of oxygen as it occurs in real tissue.

In addition, it is difficult to imagine that laboratory meat producers will be in a position in the near future to offer consumers a wide range of meats reflecting the diversity of animal muscles or cuts. Indeed, the sensory quality (i.e., flavor) of meat differs across species (pork, poultry, ovines, bovines, etc), and within a species, between breeds, genders, animal types (i.e., young bulls, steers, heifers, and cows in the case of bovines), farming conditions (depending for instance on breeding location), and mainly between muscles with a different anatomic location (22). So, many complex processes still need to be controlled to make *in vitro* meat more attractive to consumers as it is more or less the case for any other new food product.

### Health and Safety

Advocates of *in vitro* meat claim that it is safer than conventional meat, based on the fact that lab-grown meat is produced in an environment fully controlled by researchers or producers, without any other organism, whereas conventional meat is part of an animal in contact with the external world, although each tissue (including muscles) is protected by the skin and/or by mucosa. Indeed, without any digestive organs nearby (despite the fact that conventional meat is generally protected from this), and therefore without any potential contamination at slaughter, cultured muscle cells do not have the same opportunity to encounter intestinal pathogens such as *E. coli, Salmonella* or *Campylobacter* (10), three pathogens that are responsible for millions of episodes of illness each year (19). However, we can argue that scientists or manufacturers are never in a position to control everything and any mistake or oversight may have dramatic consequences in the event of a health problem. This occurs frequently nowadays during industrial production of chopped meat.

Another positive aspect related to the safety of cultured meat is that it is not produced from animals raised in a confined space, so that the risk of an outbreak is eliminated and there is no need for costly vaccinations against diseases like influenza. On the other hand, we can argue that it is the cells, not the animals, which live in high numbers in incubators to produce cultured meat. Unfortunately, we do not know all the consequences of meat culture for public health, as *in vitro* meat is a new product. Some authors argue that the process of cell culture is never perfectly controlled and that some unexpected biological mechanisms may occur. For instance, given the great number of cell multiplications taking place, some dysregulation of cell lines is likely to occur as happens in cancer cells, although we can imagine that deregulated cell lines can be eliminated for production or consumption. This may have unknown potential effects on the muscle structure and possibly on human metabolism and health when *in vitro* meat is consumed (21).

Antibiotic resistance is known as one of the major problems facing livestock (7). In comparison, cultured meat is kept in a controlled environment and close monitoring can easily stop any sign of infection. Nevertheless, if antibiotics are added to prevent any contamination, even occasionally to stop early contamination and illness, this argument is less convincing.

Moreover, it has been suggested that the nutritional content of cultured meat can be controlled by adjusting fat composites used in the medium of production. Indeed, the ratio between saturated fatty acids and polyunsaturated fatty acids can be easily controlled. Saturated fats can be replaced by other types of fats, such as omega-3, but the risk of higher rancidity has to be controlled. However, new strategies have been developed to increase the content of omega-3 fatty acids in meat using current livestock farming systems (23). In addition, no strategy has been developed to endow cultured meat with certain micronutrients specific to animal products (such as vitamin B12 and iron) and which contribute to good health. Furthermore, the positive effect of any (micro)nutrient can be enhanced if it is introduced in an appropriate matrix. In the case of *in vitro* meat, it is not certain that the other biological compounds and the way they are organized in cultured cells could potentiate the positive effects of micronutrients on human health. Uptake of micronutrients (such as iron) by cultured cells has thus to be well understood. We cannot exclude a reduction in the health benefits of micronutrients due to the culture medium, depending on its composition. And adding chemicals to the medium makes cultured meat more “chemical” food with less of a clean label.

### Comparison of Environmental Impact With Conventional Farming

Generally speaking, the production of cultured meat is presented as environmentally friendly, because it is supposed to produce less GHG (which is a matter of controversy), consume less water and use less land (this point being obvious) in comparison to conventional meat production (13, 24, 25), from ruminants particularly. However, this type of comparison is incomplete and sometimes biased or at least, partial as discussed below.

Regarding GHG, it is true that livestock, mainly ruminants (i.e., cattle), are responsible for a significant proportion of world GHG emissions, in large part due to methane emissions from the digestive tracts of herbivores. As such, reducing methane emissions (one of the most potent GHG) is presented as one of the more important potential benefits of *in vitro* meat over conventional livestock farming. Cattle farming is, as well-known, associated with the emission of three GHG [especially methane (CH_4_), but also carbon dioxide (CO_2_), and nitrous oxide (N_2_O)]. On the contrary, emissions by cultured meat are mainly CO_2_ due to fossil energy use to warm cultured cells. Nevertheless, in carbon equivalent, there is no consensus about GHG emissions of lab-grown meat compared to conventional meat: a first study gave an advantage to cultured meat (25) whereas a second study was inconclusive (26).

In a recent study, Lynch et al. (24) concluded that global warming will be less with cultured meat than with cattle initially, but not in the long term because CH_4_ does not accumulate as so long in the atmosphere unlike CO_2_. In some cases, cattle systems are characterized by a greater peak warming compared to *in vitro* meat. However, their warming effect will decline and will be stabilized with the new emission rates of cattle systems. On the other hand, warming due to the long-lived CO_2_ gas from *in vitro* meat will persist. It will even increase with a low meat consumption, being even higher than that of cattle production in some cases. They concluded that the potential advantage of cultured meat over cattle regarding GHG emissions is not obvious.

Otherwise, some scientists (27) demonstrated that conventional beef production systems in the USA (finished in feedlots with growth-enhancing technology), produce less GHG emissions, and require the fewest animals, water, and land, with a relatively low carbon footprint to produce beef, compared to a -fed systems. Indeed, with the shortest time interval from birth to slaughter, conventional systems require less maintenance energy.

So, the respective impacts of cattle and cultured meat will depend on the availability of systems for energy generation and of production systems that will be in place.

Regarding water consumption, it is claimed in the media that 15,000 L of fresh water are necessary to produce 1 kg of beef. In reality, 95% of this amount of water is used for the growth of crops, plants and forages to feed animals. Much of this water is not saved if farm animals are removed from pastures and land. Thus, different methods give wildly different results for the same livestock product. It is now accepted that the production of 1 kg of beef will require 550–700 L of water as reviewed some years ago (28, 29). This reference point is important for the comparison of water requirements for the production of cultured meat. Unfortunately, the comparison was unfair because it was on 15,000 L. It should be based on 550–700 L. One other issue is the quality of water, which may be not so good from cultured meat factories, if we consider the activities of the chemical industry for the production of the growth factors and hormones required for cell culture. Indeed, waste and spillage of chemical products could occur and these products may be in water discharged into the environment by meat incubators, which is, however, unlikely to occur in highly controlled circumstances.

Regarding land, it is obvious that cultured meat will need less land than conventional meat production, largely based on pasture. However, this does not equate to an advantage for cultured meat. Indeed, livestock plays a key role in maintaining soil carbon content and soil fertility, as manure from livestock is a source of organic matter, nitrogen, and phosphorus. Furthermore, while it is true that the production of feed for farm animals requires 2.5 billion ha of land (i.e., about 50% of the global agriculture area), 1.3 billion ha (of land used for feed production) corresponds to non-arable grasslands, useable for livestock only (30).

Land use is a distorted and unfair comparison between cultured meat and conventional meat. Indeed, in this type of comparison, authors do not take into account the diversity of environmental services and impacts of livestock farming systems (not only GHG emissions and water use, but also carbon storage and biodiversity of plants and of animals as well) (4, 31).

### Comparison of Welfare Issues With Conventional Farming

Animal welfare is a major focus of concern in some parts of our modern society. For example, Mark Post observed that there is an increasing trend of awareness of animal welfare among the Western community (16). Therefore, there are some animal defenders who can readily accept the concept of cultured meat and some have labeled cultured meat as “victimless meat” (32). Despite the fact that the process of cultured meat needs muscle samples from animals, the number of slaughtered animals can be reduced significantly (33).

However, nowadays, issues of animal welfare concern mainly cattle feedlots and pig and poultry industrial production units. Indeed, with their very high animal concentrations and associated economies of scale, such industrial units also compete strongly with smallholder farms, which are declining worldwide.

In addition, if livestock are removed and replaced with cultured meat, a number of livestock services will be lost. Indeed, livestock farming systems perform numerous functions: besides supplying proteins for human nutrition, livestock provide income for rural populations and thus support a large part of the world's rural communities. Livestock produce not only meat, milk, and eggs, but also wool, fiber, and leather. They also provide socio-cultural services including tourist events such as transhumance, and products with a local image and sense of *terroir* such as Protected Designation of Origin cheeses and other products (4, 31).

## Market and Legislation

A recent review (34) detailed (i) the market for cultured meat, and (ii) identified key consumer, political, and regulatory issues for cultured meat.

### Market

The first *in vitro* hamburger was made in 2013 after 2 years in development, by Professor Mark Post from Maastricht University. The price of this innovation was more than $300,000 in 2013. This high cost was explained by the fact that Professor Post used products and compounds (such as hormones and nutrients) traditionally used in medical science. Soon after the presentation of this innovation, Professor Post received further investments and founded a team of researchers to develop *in vitro* meat within a new start-up called *Mosa Meat*. Today, he is suggesting that in 2021, the same hamburger will be worth around US$9, which is still expensive compared to the regular hamburger at $1 (35). Furthermore, *Mosa Meat* has recently announced the development of serum-free medium according to their website's FAQ (36). No cultured meat has yet to reach the stores' shelves and the project needs more research to lower its price.

Livestock farmers are worried about the steady progress made by the aforementioned research. Indeed, the potentially effortless and low-cost production of *in vitro* meat is supposed to make it more economical than regular meat. Moreover, the issue of spoilage and of pathogens are different between cultured meat and conventional meat: keeping contamination out of cultured meat is going to be a challenge when manufacturing is scaled up and one is using a factory and not a laboratory.

Among the solutions, cultured meat is presented as a good alternative (37, 38) for consumers who want to be more responsible but do not wish to change the composition of their diet (10–13).

A recent survey shows that a potential consumer of cultured meat (which is in development) is described as a young, highly educated meat consumer, who is a little familiar with *in vitro* meat and willing to reduce their slaughtered meat consumption (39).

Due to the rise in demand for protein analogs, cultured meat sales may increase in the near future (34). Indeed, some researchers consider this new meat as a vegetarian product—good news for the expanding number of consumers who are incorporating more vegetarian and vegan choices into their diets (40, 41).

For example, Informa Agribusiness Intelligence estimates that by 2021, UK sales of meat analogs will grow by 25% and milk alternatives by 43%; such growth will take the total UK sales of milk alternatives from £149 million (US $208 million) to £299 million (US$400 million) (34). In fact, cultured meat start-ups, as well as farmhouse cheesemakers and charcuterie producers, will have a wide range of opportunities to create their own product version, leading to additional brand diversity and competitiveness in the market, as well as engaging in higher skilled jobs in a new knowledge economy (34).

In addition, different studies have shown that acceptance of cultured meat will vary substantially across cultures (42), between gender (43) and depending on the amount of provided information about cultured meat (43). Moreover, as said previously, cultured meat is one of the solutions presented as a good alternative for consumers who want to be more responsible, but do not wish to change the composition of their diet.

As with any food product, consumers will not be willing to accept any compromises in terms of food safety or indeed to compromise much on taste or other attributes (42). Indeed, consumers are still highly influenced by the sensory quality of meat. Thus, plant-based meat alternatives have been developing and have improved a lot in terms of sensory traits in recent years, because a lot of progress has been made in mimicking real meat. Therefore, with high sensory/organoleptic quality, these meat substitutes should not be considered as an intermediate step leading to the acceptance and greater consumption of artificial meat. Indeed, sales of meat analogs made from plant-based proteins and mycoproteins may increase more than cultured meat in the near future. These meat substitutes are holding an important market share (19, 43), especially in light of the fact that $16 billion was invested in start-ups and companies offering vegetable meat substitutes ($673 million in 2018), which is much more than investments in start-ups working on cultured meat (about 100 to 200 million since 2015). Therefore, some scientists consider that cultured meat is already obsolete since progress in plant-based meat alternatives is already well advanced (44).

**Figure 1:**
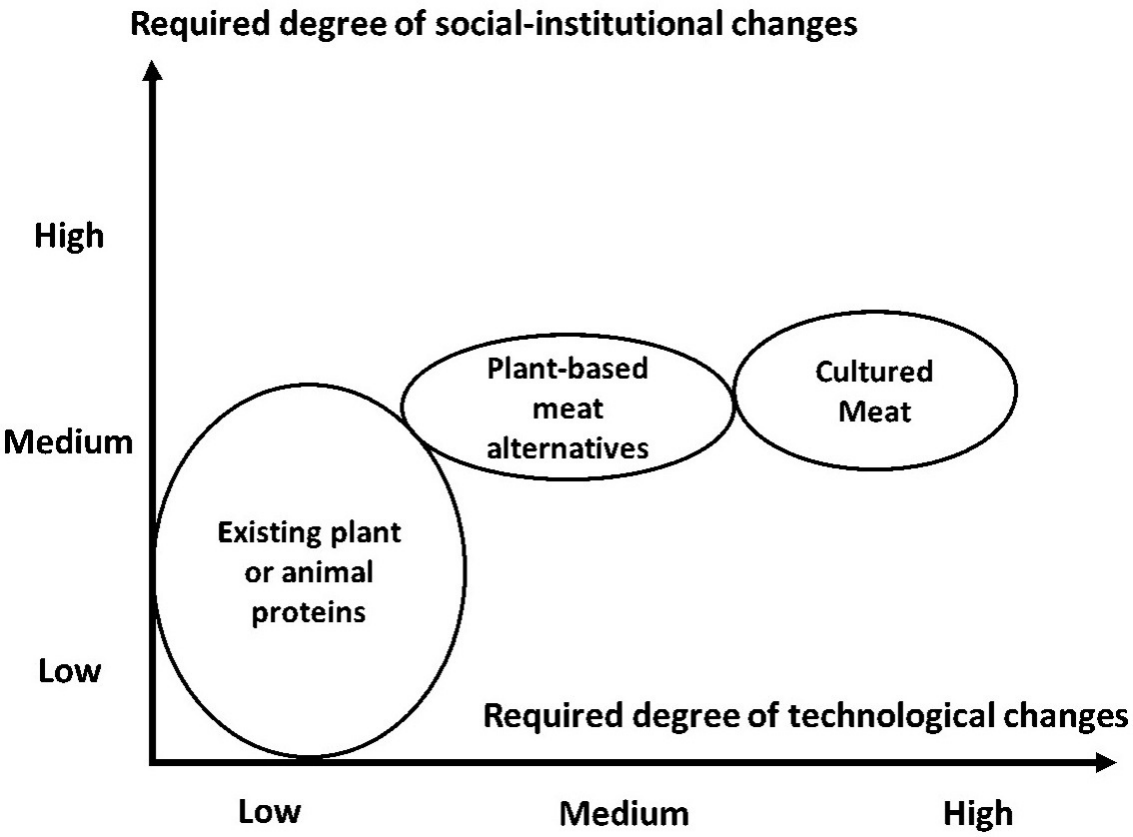
Degree of social-institutional and technological change required for meat alternatives. Adapted from Van der Weele et al. (38).

Furthermore, the meat industry of the future will undoubtedly be more complex than the meat industry today, with a greater number of meat products or meat substitutes on the market coming from different sources or processes (19, 43). All protein sources inherently contain both drawbacks and advantages that will affect their ability to be commercialized and accepted by consumers (43). For new products to be successful, they must be commercially viable alternatives to conventional meat production. The success of cultured meat as an alternative, substitute or complement to conventional meat will play an important role, because consumers are likely to refer to products with similar positioning in the market (38, 42, 45). Indeed, if the palatability issues are solved (which is the case today with at least some plant-based meats) and if meat substitutes are competitive in terms of price, consumers will be more open to changing their purchasing habits (43, 46, 47). However, the most technologically challenging alternatives to meat also require moderate to high degrees of social-institutional change (38). A recent study conducted by Van der Weele et al. (38) demonstrates that cultured meat and plant-based meat alternatives both require a moderate degree of social-institutional change (from the current Western dietary patterns), even if they don't require the same degree of technological change, given that, unlike cultured meat, some plant-based products are already being commercialized (Figure 1). In brief, to be successful, new beef products (either from the conventional beef industry or from the “*FoodTech*” industry) will need to be competitive and sustainable and in keeping with consumption habits and cultural models.

Indeed, cultured meat requires a high degree of technological change, which may compromise a rise in its consumption. On the other hand, plant-based proteins are present in some products that are already commercialized. Some existing protein sources are either well accepted (beef, pork, meat from poultry, crops, etc.), whereas others are much less consumed or accepted (such as meat from horses, guinea pigs etc.), despite their consumption in some countries.

### Legislation

A small but important body of literature exists on the regulation of cultured meat, with Schneider (48) considering regulation in the United States and Petetin (49) considering regulation in the European Union (34).

In terms of status, *in vitro* meat stands at the frontier between meat and non-meat. In April 2018, France had already banned the use of meat- and dairy-related words to designate vegetarian and vegan products. The use of the word “meat” for *in vitro* meat has not been decided yet (50). Livestock farmers in the US are backing a new law in Missouri, which states that for a product to be called “meat,” it has to come from a real animal as indicated in most dictionaries. Furthermore, meat scientists differentiate between “muscle” and “meat,” with the latter being the result of a natural biological process of muscle aging after slaughter due to the cessation of oxygen supply to muscle cells (51). Should “cultured meat” be called meat? If not, should *in vitro* meat still be regulated in the same way as regular meat? (52).

It is likely that the response on regulation will take time, and it is possible that the definition of “meat” will vary between countries. The Cattle Council of Australia CEO, Margo Andrae, is already warning “cultured meat companies” to avoid repeating a battle over terms as happened with “milk” and “dairy”; her view is that it should “be called what it is, which is lab-grown protein” (50). Furthermore, the various start-ups have clearly different strategies based on marketing choices, with some of them calling the product “animal protein” and others “artificial meat.” The former are driven by the will to tell the truth to consumers, the latter by a desire to be provocative in order to increase consumer interest (43).

## Public Perception

How consumers perceive and accept or reject cultured meat is largely a matter of controversy (42, 53).

### Consumer Perception

Advocates of cultured meat are concerned that the name could put off consumers, with possible connotations of a product that is “fake.” Indeed, the lack of consumer acceptance could be a major barrier to the introduction of cultured meat (54). Furthermore, it seems difficult to evaluate consumer acceptance for an earlier stage product, which does not exist yet, as cultured meat.

It is widely acknowledged that the name given to an object or phenomenon can affect subsequent evaluations and impressions of it. In this way, different names which have an influence on consumer attitude were proposed for cultured meat (55, 56). Indeed, “*in vitro* meat,” “clean meat,” “cultured meat,” “lab-grown meat,” “synthetic meat” and other names (15) suggest that this innovation is slaughter-free, more responsible toward our environment and a credible alternative to the current intensive farming systems.

Otherwise, some authors have demonstrated (57) that consumers tend to strongly reject the name “*in vitro* meat.” Moreover, the term “cultured” is less disliked than the terms “artificial” and “lab-grown” (57). This is confirmed by the Siegrist et al. study (54), which concluded that participants have a low level of acceptance of cultured meat because it is perceived as unnatural. Furthermore, they found out that giving information to participants in the survey about the production of cultured meat and its benefits has the paradoxical effect of increasing the acceptance of traditional meat (54). Bryant et al. (58) and Siegrist and Sütterlin (59) argued that a higher acceptance may be favored by less technical descriptions of cultured meat. This may be explained by the fact that the “high- tech” process is associated with something scientific and unnatural, and therefore negatively affects the product's image. In reality, consumers seem to dislike unnatural food.

In the study of Verbeke et al. (42), conducted in three EU countries, researchers demonstrated that “consumers' initial reactions when learning about cultured meat were initially underpinned by feelings of disgust and considerations of unnaturalness. After thinking, consumers envisaged few direct personal benefits from cultured meat, but they acknowledged possible global societal benefits. Perceived personal risks from eating cultured meat were largely underpinned by considerations of unnaturalness and uncertainty, and therefore inducing some kind of fear of the unknown.” Later on, consumers may accept scientific progress and therefore cultured meat, but will require a trusted process of control and regulations to ensure complete safety of the product.

In a recent survey, Bryant et al. (58) asked participants from the USA, India and China about their willingness to try occasionally or to buy cultured meat regularly, to eat cultured meat instead of conventional meat or plant-based meat substitutes. Willingness to try or to eat cultured meat was quite high: 64.6% of the participants being willing to try it, and 49.1% willing to buy it regularly and eat it instead of conventional meat (48.5%). The authors interpreted those results in favor of cultured meat, saying that this “indicates a substantial potential market for cultured meat” with the consequences that cultured meat could replace a significant amount of conventional meat according to Bryant et al. (58). However, this contradicts the results of a survey by Hocquette et al. (60), who found that the majority of more educated consumers from different countries will not buy cultured meat regularly although one-third of the respondents answered “I do not know.” Moreover, consumers' vision of cultured meat is likely to change over time through receiving more information.

### Ethics

Ethical issues are more and more important in food choices (61), and this encourages the development of social or societal concerns (21). While the potential advantages of cultured meat regarding ethics and environmental issues are acknowledged, many consumers have concerns about food safety mainly due to the unnaturalness perception of cultured meat (42, 53) as discussed previously.

*In vitro* meat, like any new technology, raises inevitable ethical issues. One of the main purposes of this innovation, according to cultured meat advocates, is to stop the cruel practices endured by animals that are sometimes confined in tight spaces and slaughtered in inhumane conditions. Besides, the usual conditions of life for battery-farmed animals often lead to diseases, infections, behavioral problems, and suffering. However, due to the lack of a nervous system, cultured cells and *in vitro* meat are supposed to be free from any type of pain (62, 63) although biopsies on animals to collect cells may raise some issues concerning animal welfare. Therefore, some scientists consider this new (artificial) meat as a vegetarian product (62, 64, 65).

Thus, cultured meat aims to use considerably fewer animals than conventional livestock farming. Indeed, from an animal welfare perspective this could be attractive to some vegetarians, vegans and those conscientious omnivores interested in reducing their meat intake for ethical reasons (64).

The aforementioned idea would be more accurate if, as some start-ups have claimed, a new type of medium has been developed without the use of FBS from dead calves. Actually, some vegans have been avoiding animal food because of the meat taste. Others would consider eating it if it was produced in a cruelty-free and friendly environment (66).

Otherwise, while many scientific authors recognize the potential ethical benefits of artificial meat, namely an increase in animal welfare, nutrition-related diseases, food-borne illnesses, resource use, and greenhouse gas emissions (32), other authors, as discussed previously, are not convinced that the production of artificial meat will have a low carbon footprint. Nevertheless, it is clear that the environmental impact of artificial meat is difficult to evaluate because it is currently based on speculative analyses (21).

But it is not that simple. There are certain issues to be considered. For example, at present, animals still have to be used in the production of cultured meat, even in fewer numbers for muscle sampling only. Whether painful or painless, animals must be reared so that their cells can be harvested to produce *in vitro* meat. “Consequently, lab-grown meat still involves animal exploitation, which is what the proponents of artificially grown meat want to avoid” (66).

### Naturalness

However, if this description is true for some intensive livestock systems, whereas intensive livestock remains cruel for a lot of people, it is not the case for a significant proportion of livestock in the world, and particularly for many extensive systems in France or some African countries. In a recent review, some authors (67) concluded that sustainable intensification and agroecology could converge for a better future by adopting transformative approaches in the search for ecologically benign, socially fair and economically viable livestock farming systems.

### Religion and Meat Consumption

*In vitro* meat, like any other new technology, raises numerous ethical, philosophical and religious questions. Mainly because of its nebulous status, religious authorities are still debating the following: whether *in vitro* meat is *Kosher* (consumable under Jewish dietary laws), *Halal* (for Muslim consumers, compliant with Islamic laws), or what to do if there is no animal available for ritual practices (Hindu consumers).

Concerning the Jewish religion, rabbinical opinion is divided. Some think that cultured meat can only be considered *Kosher* if the original cells were taken from a slaughtered *Kosher* animal. Others assume that regardless of the source of the cells used to produce the cultured meat, they will certainly lose their original identity. Therefore, the outcome cannot be defined as forbidden for consumption (68).

For the Islamic community, the crucial question is whether the cultured meat is compliant with Islamic laws or not, most commonly referred to as “*Halal* or not.” Since meat culturing is a recent invention, the traditional Islamic jurist that Muslims often refer to has never discussed its *Halal* status. Therefore, contemporary Islamic jurists have taken on this mission. The *Halal* status of cultured meat can be resolved through identifying the source of the cells and serum medium used in culturing the artificial meat. Accordingly, *in vitro* meat is considered *Halal* only if the stem cell is extracted from a *Halal* slaughtered animal, and neither blood nor serum is used in the process. Indeed, serum should be avoided unless one can prove that the meat will not be changed as a result of contact with the serum (being potentially unclean) (14).

## Conclusion

To meet the increasing demand for food by a growing population in 2050, the FAO has concluded that 70% more food will be needed to fulfill this demand. In this context, livestock systems will be a vital element in addressing global food and nutrition security in the world. However, to avoid criticism of livestock farming concerning environmental and animal welfare issues, more efficient ways of protein production are being developed to sustain the growing global population.

One option is to culture muscle cells in an appropriate culture medium, the most efficient so far being a medium containing FBS. The medium should provide nutrients, hormones, and growth factors, so that muscle cells will proliferate before being converted into muscle and hence produce a huge amount of meat from a limited number of cells. Hopefully, thanks to technical advances, FBS has been replaced, at least in research laboratories, but maybe not yet at the industrial level. Furthermore, as hormone growth promoters are prohibited in conventional farming systems for conventional meat production in the European Union, this is still an issue. However, this technique is able to produce disorganized muscle fibers which are far removed from real muscle, and this is a huge limitation in seeking to reproduce the wide range of meats representing the diversity of animal species and breeds, as well as muscles or cuts. Moreover, the role of blood vessels and blood, nerve tissue, intramuscular fats, and connective tissue affect both taste of meat. Indeed, a number of the “good” veggie meat burgers fail on texture and taste from the point of view of being too uniform.

The nutritional quality of cultured meat can be theoretically controlled by adjusting the fat composites used in the medium of production. This is also the case with conventional meat, with newly-developed strategies increasing the content of omega-3 fatty acids in meat with current livestock farming systems. However, controlling the micronutrient composition of cultured meat is still a research issue. Finally, the impact of cultured meat consumption on human health will have to be carefully checked and documented.

Regarding GHG, there is no consensus on the potential advantages in terms of GHG emissions of lab-grown meat compared to conventional meat on a short-term or long-term basis.

Despite its current high price, the production costs of cultured meat will probably decrease in the near future. This may help consumer acceptance, despite a strong rejection of names that refer to “*in vitro*” or “cultured” meat technology. However, cultured meat will be in competition with other meat substitutes already on the market and better accepted by consumers, such as plant-based products.

Ethically, cultured meat aims to use considerably fewer animals than conventional livestock, which makes the product attractive to vegetarians and vegans. However, a few animals will still need to be reared so that their cells can be harvested to produce *in vitro* meat.

Moreover, the religious authorities are still debating; whether *in vitro* meat is *Kosher* (consumable under Jewish dietary laws), *Halal* (for Muslim consumers, compliant with Islamic laws).

In conclusion, it seems clear that research projects on cultured meat have had a limited scope as *in vitro* meat development is still in its infancy. The product will evolve continuously in line with new discoveries and advances that optimize the production, quality and efficiency of cell division. It remains to be seen whether this progress will be enough for artificial meat to be competitive in comparison to conventional meat and the increasing number of meat substitutes.

## Author Contributions

SC and J-FH contributed equally in the redaction of this review. All authors listed have made a substantial, direct and intellectual contribution to the work, and approved it for publication.

### Conflict of Interest

The authors declare that the research was conducted in the absence of any commercial or financial relationships that could be construed as a potential conflict of interest.
